# Medicines without Doctors: Why the Global Fund Must Fund Salaries of Health Workers to Expand AIDS Treatment

**DOI:** 10.1371/journal.pmed.0040128

**Published:** 2007-04-17

**Authors:** Gorik Ooms, Wim Van Damme, Marleen Temmerman

## Abstract

Using the example of two countries, Mozambique and Malawi, which are fighting a full-blown HIV epidemic with a fragile health system, the authors discuss the crucial role of Global Fund support to the health workforce.

The Global Fund to Fight AIDS, Tuberculosis and Malaria (“the Global Fund”) was created to fight three of the world's most devastating diseases. Since its creation in 2002, it has struggled with the difficult task of focusing on three diseases, and at the same time supporting the fragile public health systems that are supposed to implement this fight on the ground.

Recent internal comments from the Global Fund suggest an intention to focus more on the three diseases, and to leave the strengthening of health systems and support for the health workforce to others. This could create a “Medicines without Doctors” situation in which the medicines to fight AIDS, tuberculosis, and malaria are available, but not the doctors or the nurses to prescribe those medicines adequately.

We believe that this would be a strategic mistake, as the Global Fund has an advantage that makes it a key actor in the field of supporting health workforces. Most other donors are forced to aim for sustainability in the conventional sense (implying that beneficiary countries should gradually replace international funding with domestic resources); the Global Fund has been promised sustained funding by the international community, allowing it to make sustained commitments to beneficiary countries. This is what some of the countries most affected by AIDS, tuberculosis, and malaria need to increase their health workforce. Their health workforce challenges are too big to consider a gradual replacement of international funding with domestic resources.

We also believe that the debate about this intention should be public, and we hope to launch the public debate with this article.

We use the examples of two countries—Mozambique and Malawi—trying to fight against a full-blown AIDS epidemic with a fragile health system, to underline the crucial role of Global Fund support to the health workforce.

## The Health Workforce Gap in Mozambique

The World Health Organization estimates that to achieve the Millennium Development Goals (MDGs), health systems need at least 2.5 health workers per 1,000 people [1]. In Mozambique, there are 514 doctors, 3,954 nurses, and 2,229 midwives: per 1,000 people there are 0.36 full-time equivalents of health workers (2004 figures) [2]. Mozambique's health workforce would have to be multiplied by seven to achieve the MDGs.

To roll out antiretroviral therapy (ART) across the country, Mozambique estimates that it would need eight health workers per 1,000 patients receiving ART [3]. This is in line with the estimations of Hirschhorn et al.: the numbers of health workers required to provide ART to 1,000 patients include one to two physicians, two to seven nurses, one to three pharmacy staff, and a wide range of counsellors and treatment supporters [3]. These findings apply to ART programmes in their start-up phase, which require an intensive follow-up, but even if a mature ART programme could be effective with only four health workers per 1,000 patients, the number of additional health workers required remains a huge challenge, knowing that 199,000 people in Mozambique needed ART by the end of 2005 [4].

Is Mozambique's health workforce gap exceptional? There are 12 countries in Africa with an HIV prevalence of more than 5% and less than two nurses per 1,000 people (see Table 1). If we rank these countries according to density of nurses, Mozambique comes last. In terms of expanding access to ART, no country faces a bigger health workforce crisis than Mozambique.

**Table 1:**
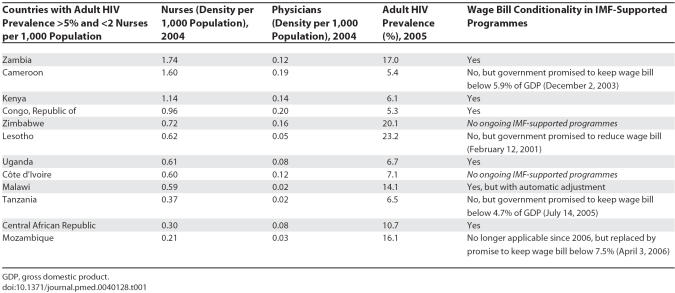
Health Workforce Gaps and Wage Bill Agreements in 13 African Countries

## The Health Workforce Gap in Malawi

In Malawi, there are 266 doctors and 7,264 nurses (no figures on midwives are available): per 1,000 people there are 0.61 full-time equivalents of health workers (2004 figures) [5]. The health workforce would need to be multiplied by four to achieve the MDGs.

In 2004, Peter Piot, head of the Joint United Nations Programme on HIV/AIDS, and Suma Chakrabarti, permanent secretary of the United Kingdom Department for International Development, during a joint visit to Malawi concluded that it would be impossible to roll out ART without undermining the health system, unless the level of health workers could be increased dramatically. They instructed their agencies to support an initiative to address the health workforce crisis. The result was “a shift from piecemeal donor support for a number of uncoordinated initiatives to a more comprehensive approach” [6].

The response in Malawi might remain unique for several reasons. First, the Malawi response was possible because of an explicit decision by donors “to consider measures that might otherwise be dismissed as unsustainable” because of the scale of the crisis [6]. It is not clear how serious a health workforce crisis needs to be for donors to consider “unsustainable” measures.

Second, Malawi was able to come to a special agreement with the International Monetary Fund (IMF). Malawi agreed to a ceiling on the “government wage bill” with the IMF in September 2003. In July 2005, the IMF accepted that the ceiling “will be adjusted upward ([or] downward) by the full amount of donor-funded supplementary wages and salaries for the health sector that is greater ([or] less) than the program baseline” [7]. All countries listed in Table 1 have agreed with the IMF to control their wage bill—either as a performance criterion or benchmark, or as a promise in a “Letter of Intent”—except for Zimbabwe and Côte d'Ivoire, which do not have ongoing IMF-supported programmes. Malawi is the only country benefiting from an automatic adjustment of this ceiling. The IMF justifies these ceilings because of “concerns about potential macroeconomic problems that could result from entering into long-term expenditure commitments without long-term donor commitments to finance them” [8].

In addition, Malawi obtained funding from the Global Fund under its Fifth Call for Proposals. The Board of the Global Fund decided to consider health systems strengthening (HSS) interventions for funding as a specific category under its Fifth Call for Proposals, and it was as an HSS intervention that the Malawi response was approved. But under the Sixth Call for Proposals, specific HSS interventions were no longer eligible.

## Global Fund Support to the Health Workforce

The Global Fund has a unique governance structure. At the core of this structure are the Country Coordination Mechanisms (CCMs): national platforms of stakeholders, formulating proposals in answer to the calls for proposals launched by the Board of the Global Fund. (The Board of the Global Fund regularly launches calls for proposals, known as Rounds: Round 1 and Round 2 were launched in 2002, Round 3 in 2003 and so forth. Round 7 was launched in March 2007.)

These proposals are reviewed by the Technical Review Panel (TRP), a panel of independent experts. The TRP recommends certain proposals for funding to the Board.

The Global Fund's Board includes representatives of donor and recipient governments, non-governmental organisations, the private sector, and affected communities. It approves proposals upon recommendation from the TRP. It also approves the guidelines and the proposal forms for each of the Rounds of the Global Fund.

The Secretariat is the executive branch of the Global Fund. In principle, it does not interfere with the approval process. In practice, it does elaborate the guidelines and the proposal forms, and thus it has an influence on the eligibility of proposals.

For an intervention to be eligible, it needs to be proposed by a CCM, recommended by the TRP, approved by the Board, and it must fit within the guidelines and proposal forms proposed by the Secretariat.

As an illustration of the complexity of this governance structure, we could mention the initial uncertainty about the eligibility of AIDS treatment interventions. During the first Board meeting, the Health Minister of France said that “there should be no false dilemma over treatment or prevention”, but did not receive a clear answer from the Board [9]. Then CCMs proposed ART interventions, the TRP recommended some of them, and the Board approved them.

By doing so, the Global Fund has developed—perhaps implicitly—a novel approach to sustainability. Sustainability in the conventional sense implies that beneficiary countries gradually replace foreign assistance with domestic resources. This is not realistic for low-income countries providing ART. Nonetheless, the Global Fund does support ART interventions in low-income countries: thus it shifted concerns about sustainability from national to international level (if the Global Fund can sustain these interventions, they are sustainable, albeit in a different manner).

The international community endorsed this novel approach. In June 2006, the United Nations General Assembly committed itself “to supporting and strengthening existing financial mechanisms, including the Global Fund to Fight AIDS, Tuberculosis and Malaria, as well as relevant United Nations organizations, through the *provision of funds in a sustained manner*” (emphasis added) [10]. It might sound like a nuance, but the difference between “sustainability relying on domestic resources in the long run” and “sustainability relying on the provision of external funds in a sustained manner” is fundamental.

This novel approach is what countries like Mozambique need to strengthen their workforce. They need to hire more health workers, but they are unable to sustain the costs of hiring additional health workers with domestic resources.

If the controversy about ART was easy to solve, the controversy about strengthening health workforces was tougher. Some Global Fund supporters understood from the beginning that its success in expanding coverage of ART depended on its willingness to pay for the salaries of additional health workers. However, the Global Fund has never been keen to expand its novel approach to sustainability to the funding of the health workforce. Since Round 2, the Global Fund has applied strict criteria for the funding of salaries of health workers. With regards to salaries, applicants must explain “how these salaries will be sustained after the proposal period is over” [11]. Dräger et al. note that this concern about sustainability “cannot be found for any other activities financed by the Global Fund” and suspect that it is closely linked to IMF and World Bank macroeconomic policies [11].

The advocates of supporting salaries of health workers from the Global Fund obtained a short-lived victory in 2005, when Round 5 of the Global Fund included a specific category for HSS interventions.

But Round 5 also caused some actors to evaluate their role in the global health landscape. The World Bank insisted on a “Comparative Advantage Study” of Global Fund and World Bank AIDS programmes. Alexander Shakow, who conducted the study, recommended that the Global Fund focus on disease-specific interventions, leaving HSS interventions to the World Bank [12]. In January 2006, the Global AIDS Alliance and Health GAP—supported by more than 30 experts and 300 non-governmental organisations—urged the Global Fund to keep HSS interventions as a specific category [13,14]. In April 2006, the Board decided to narrow the scope of eligible interventions, adopting a proposal form that no longer included HSS interventions as a specific category. In August 2006, Richard Feachem, the executive director of the Global Fund, endorsed a new “division of labour” between the World Bank and the Global Fund: rapid scale-up of disease-specific programmes for the Global Fund and long-term development of health infrastructure for the World Bank [15]. In November 2006, the TRP and the Secretariat, in their report to the Board, recommended that “the Board convene a suitable forum, which can discuss and attempt to resolve the question of the appropriate scope and definition of acceptable HSS activities prior to Round 7. Ideally, this discussion will lead to a clarification and narrowing of the scope of HSS activities which the Global Fund sees as its mandate to fund” [16].

This evolution is problematic because the World Bank does not share the Global Fund's novel approach to sustainability, certainly not for health workers' salaries. The World Bank believes that “it is not prudent for countries to commit to permanent expenditures for such items as salaries for nurses and doctors on the basis of uncertain financing flows from development assistance funds” [17].

Some bilateral donors might be willing to consider “unsustainable” interventions to address health workforce crises, as they did in Malawi. But Malawi remains the exception that confirms the general rule. Bilateral donors will find it difficult to make their commitments reliable enough for the IMF to adjust the ceiling on the government wage bill. Most bilateral donors can only commit for as long as their government remains in place—only a few years.

## Conclusion

Both the cases of Mozambique and Malawi illustrate the crucial importance of addressing the health workforce crisis. It is easier to remedy the shortage of medicines with external funding than it is to remedy the shortage of health workers with external funding. Medicines can be bought; health workers need to be trained first. This underlines the importance of starting emergency human resources programmes now, before the growing case load—resulting from the fact that most people on ART will stay alive longer, while the number of people in need of ART will grow—undermines either the quality of ART programmes, or the performance of health systems [18].

Without support from the Global Fund, it will be difficult for Mozambique to develop its own emergency human resources programme. Bilateral donors are unable to support human resources programmes that rely on sustained external assistance over decades. The World Bank is unwilling to use foreign assistance for salaries of health workers. The IMF is unwilling to stretch ceilings on wage bills, because commitments from bilateral donors are unreliable. Without flexibility about these ceilings, bilateral donors cannot support salaries of doctors and nurses, even if they want to. It is a vicious circle.

The Global Fund is probably the only actor able to break through this vicious circle. It is the only donor mechanism that benefits from an explicit endorsement from the international community to practice a novel approach to sustainability.

But donors must give the Global Fund the resources to do so. As one of us argued in a previous article, it is feasible to turn the Global Fund into a world health insurance, funded by rich countries in accordance with their wealth, and creating rights for poor countries to obtain assistance in accordance with their needs [19]. It would allow individual donors to overcome their inability to make commitments beyond the term of their governments, because their contributions would be compulsory. (This is not a heresy. Many bilateral donors consider their contributions to the World Bank as compulsory [20]. This can be achieved for contributions to the Global Fund.) Furthermore, the pooling of resources by many donors would increase continuity: if one donor reduces its contribution, another donor could compensate.

And that is exactly what countries like Mozambique need to increase their health workforce: sustained assistance.

## 

**Figure:**
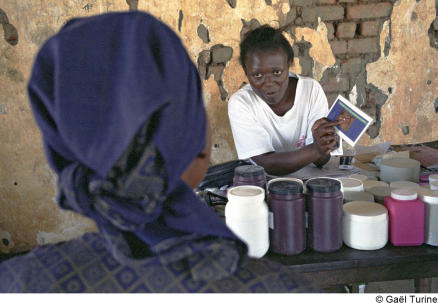
A health worker explains to a patient how to use a miconazole gum patch (Photo: Gaël Turine)

## Abbreviations

ART: antiretroviral therapy
CCM: Country Coordination Mechanism
HSS: health systems strengthening
IMF: International Monetary Fund
MDG: Millennium Development Goal
TRP: Technical Review Panel
